# Role of Prophylactic Antibiotics in Critical Care of Stroke Patients - A Preventive Approach to Post-stroke Infections?

**DOI:** 10.7759/cureus.7158

**Published:** 2020-03-02

**Authors:** Muhammad Humayoun Rashid, Ahmad Kabir, Muhammad Usman Waris, Umer Salman, Sarmad Zain

**Affiliations:** 1 Neurology, Bakhtawar Amin Medical and Dental College, Multan, PAK; 2 Internal Medicine, Nishtar Medical University and Hospital, Multan, PAK; 3 Pathology, Bakhtawar Amin Medical and Dental College, Multan, PAK; 4 Internal Medicine, Nishtar Hospital, Multan, PAK; 5 Internal Medicine, Bakhtawar Amin Medical and Dental College, Multan, PAK; 6 Internal Medicine, City Hospital, Multan, PAK; 7 Internal Medicine, Nishtar Hospital, Nishtar Medical University, Multan, PAK

**Keywords:** stroke, infection rates, prophylactic antibiotics, complications, hyperthermia, neurological outcomes, critical care, pneumonia, immunosuppression, review

## Abstract

Post-stroke complications are very common worldwide and the most common complication is infection. This contributes the most to the mortality rate in stroke patients. Among the infections, pneumonia and urinary tract infections are most common. Hyperthermia following stroke is associated with neuronal damage and worse outcomes. Post-stroke immunosuppression and activation of inflammatory mediators also cause infections. Based on the high mortality caused by post-stroke infections, various trials were done to seek the advantage that prophylactic antibiotics can give in the critical care of stroke patients. Antibiotics, including ceftriaxone (cephalosporin), levofloxacin (fluoroquinolone), penicillin, and minocycline (tetracycline), were used and the stroke patients were followed up to analyze the primary and secondary outcomes. It was concluded that early antibiotic therapy (mostly within 24 hours) leads to a reduced rate of post-stroke infections and reduced fever spikes, whereas follow-up for a longer period of time showed no better functional outcome. Furthermore, mortality and morbidity benefits were also not seen with prophylactic antibiotic therapy. This review helped us to put a nail in the coffin to the earlier thoughts that prophylactic antibiotics are necessary for the critical care of stroke patients.

## Introduction and background

A stroke occurs if the flow of oxygen-rich blood to a portion of the brain is blocked. Without oxygen, brain cells start to die after a few minutes. Sudden bleeding in the brain can also cause a stroke if it damages brain cells. Stroke is the second leading cause of death after coronary artery disease. Most of the deaths caused to stroke patients are due to infections. Among the infections, pneumonia and urinary tract infections are most common [1-2]. Hyperthermia causes cerebral injury in experimental models of focal cerebral ischemia and its harmful effect persists even if it appears days after the start of ischemia [3-5]. Hyperthermia and its association with increased mortality and morbidity in stroke patients is also well understood [6-8]. Pneumonia is believed to cause a three-fold increase in the number of deaths in post-stroke patients [9]. Infections can be caused by various factors, such as the use of invasive procedures like catheterization and mechanical ventilation [10-11]. Swallowing difficulties can lead to aspiration pneumonia [12]. Furthermore, systemic inflammatory response after stroke can cause immunosuppression and increased risk of infections [13]. It is well understood that antibiotics play a key role in treating infections. However, whether or not prophylactic antibiotics play a role in preventing post-stroke infections, and its mortality or morbidity benefits are still equivocal and under the study.

The objectives of this study were to see whether the use of prophylactic antibiotics in stroke patients reduced the risk of acute infections, was associated with better functional outcomes in follow-up visits, was associated with reduced post-stroke mortality and morbidity, and whether it reduced the length of hospital stay.

## Review

Method

Search Method

Full-text articles, including randomized control trials on the use of prophylactic antibiotics and placebo in stroke patients, were searched in PubMed, Embase, and Cochrane Library. The electronic database was searched thoroughly and requests were forwarded to a few journals for full-text availability of a few restricted articles as they fulfilled our inclusion criteria. *Inclusion Criteria*

Included articles were in the English language published prior to November 15, 2019. Randomized control trials on the use of preventive antibiotics therapy in post-stroke patients within seven days of onset were studied. The minimum age of patients included in studies was 18 years, especially those not suffering from other critical or terminal illnesses. *Exclusion Criteria* Excluded articles were cohort studies, cross-sectional studies, case reports, late use (> 7 days of stroke onset) of antibiotics, studies including patients with other critical illnesses, studies including any other therapy along with antibiotics, animal studies, studies having less number of sample size, and studies having little follow-up periods.

*Outcomes* The study included few primary and few secondary outcomes. Among the primary outcomes were the rates of early (less than 14 days) or late (greater than 14 days infection) infections, pneumonia, urinary tract infections (UTI). Among the secondary outcomes were mortality benefits, morbidity benefits, and functional outcomes on follow-ups.

*Data Collection and Analysis* This systemic review is done in accordance with the Preferred Reporting Items for Systematic Reviews and Meta-Analyses (PRISMA) guidelines [14]. Studies were randomized for the use of oral or intravenous antibiotics, the type of antibiotics used, the method of diagnosing infections, inclusion and exclusion criteria, and the sample size taken. Titles, abstracts, data analysis, and reference lists were searched for the relevant data extraction. Three reviewers independently searched various databases and collected relevant full-text articles based on inclusion criteria. Data collected included general information about the authors and the publication year, information about the characteristics of treatment and control groups, the interventions made, data about our primary and secondary outcomes, the conclusions, and the references given. Characteristics of groups included their age, sex, severity, and type of stroke and comorbidities. The intervention included the mode of antibiotics administration, the time duration of treatment, and the type of antibiotics used. Primary and secondary outcomes were as described above. Three reviewers collaborated to do the data analysis. They provided the final percentages regarding our primary outcomes. Similarly, the included trials were searched for our relevant secondary outcomes and their data was compiled. All this data was tabulated, and finally, discussions were made to reach a consensus about our final results.

Researchers studied the effects of prophylactic antibiotics and they performed various clinical trials. A few of these, which fulfilled our inclusion criteria, have been reviewed in Table 1. Among all these studies, patient sample, antibiotics used, inclusion criteria, and study design varied. Antibiotics administered were a broad-spectrum to cover most of the bugs related to pneumonia and urinary tract infections (UTIs). One study included fluoroquinolones, a few studies used minocycline, Schwarz et al. used mezlocillin, and Westrendrop et al. and De Falco et al. used ceftriaxone and penicillin [15-24]. The primary outcomes and secondary outcomes varied a little bit in all of them; a few measured the rate of infections and a few did not. Some observed long-term neurological outcomes as a part of secondary outcomes. 

**Table 1:** Summary of Different Clinical Trials Using Prophylactic Antibiotics in Post-stroke Patients IM: intramuscular; IV: intravenous; n: sample size; MCA: middle cerebral artery; N/A: not available; NIHSS: National Institute of Health Stroke Scale; RCT: randomized control trial

AUTHORS	STUDY DESIGN	INCLUSION CRITERIA	INTERVENTION	PRIMARY OUTCOMES	SECONDARY OUTCOMES	CONCLUSION
Chamorro et al. [15]	Randomized, double-blinded, placebo-controlled trial	Ischemic or hemorrhagic stroke < 24 hours, n = 136	Levofloxacin, 500 mg/100 ml/dL for 3 days, and placebo, 0.9% physiologic serum	Infection rate	Neurological outcomes and mortality benefits	No significant difference in both groups at the end of 90 days.
Schwarz et al. [16]	Randomized control trial	Ischemic stroke within 24 hours n = 60	Mezlocillin, plus sulbactam (32 g/1 g for 4 days)	Incidence and height of fever	Rate of infection and clinical outcome	Decreased body temperature, lowered the rate of infection, and may be associated with a better clinical outcome
Srivastava et al. [17]	Randomized, single-blinded, open-label study	Ischemic stroke, n = 50	Oral minocycline, 200 mg/day for 5 days, and the control group received oral vitamin B capsules	NIHSS score at 30 and 90 days	N/A	Minocycline can help to reduce the clinical deficits after acute ischemic stroke.
Kohler et al. [18]	Prospective RCT, open-label, blinded pilot study	Ischemic stroke within 24 hours, n = 95	IV minocycline, 100 mg 12 hourly for 5 doses	Survival-free of handicap at 90 days	N/A	No difference in clinical outcome at 90 days.
Amiri-Nikpour et al. [19]	Open-label, evaluator-blinded trial	Ischemic stroke within 24 hours, n = 60	Oral minocycline, 200 mg/day for 5 days	NIHSS score at 30, 60, and 90 days.	N/A	Active treatment was associated with better outcome at 90 days
Westendrop et al. [20]	Randomized, open-label trial	Ischemic/hemorrhagic, n = 2,538	IV ceftriaxone 24 hourly for 4 days	Functional outcome at 3 months	Death, Infection rates, length of hospital stay	Did not improve functional outcomes at the end of 3 months
Kalra et al. [21]	Open-label, cluster-RCT	Ischemic or hemorrhagic stroke, dysphagia, start of therapy	Antibiotic therapy (different substances) over 7 days	Pneumonia (< 14 days)	Neurological function and outcome	No difference in pneumonia and functional outcome/mortality after 90 days
De Falco et al. [22]	Open-label RCT	Ischemic stroke, inclusion	Penicillin IM	Rate of infections	Clinical outcome	Active treatment was associated with a lower rate of infections and better clinical outcome
Harms et al. [23]	Double-blinded RCT	Non-lacunar ischemic stroke, MCA territory, NIHSS > 11, start of therapy	Moxifloxacin, 400 mg over 5 days	Infection (< 11 days)		Active treatment was associated with a lower rate of infection. No difference in clinical outcome after 180 days
Lampl et al. [24]	Open-label, evaluator-blinded RCT	Ischemic stroke within 6 to 24 hours, n = 152	Minocycline, 200 mg daily for 5 days starting within 6 to 24 hours	NIHSS change from baseline to 90 days	Recurrent strokes; hemorrhagic transformation; mortality	A potential benefit of minocycline in acute ischemic stroke.

The Early Systemic Prophylaxis of Infection After Stroke (ESPIAS) study was a randomized, double-blind, placebo-controlled study of antibiotic prophylaxis in patients older than 18 years with non-septic ischemic or hemorrhagic stroke enrolled within 24 hours from clinical onset done in 2005 [15]. They observed that levofloxacin and placebo patients had a cumulative rate of infection of 6% and 6%, respectively (P = 0.96) at Day 1; 10% and 12%, respectively (P = 0.83) at Day 2; 12% and 15%, respectively (P = 0.66) at Day 3; 16% and 19%, respectively (P = 0.82) at Day 7; and 30% and 33%, respectively (P = 0.70) at Day 90. Using logistic regression, a favorable outcome at Day 90 was inversely associated with baseline National Institutes of Health Stroke Scale (NIHSS) (odds ratio (OR): 0.72; 95% confidence interval (CI), 0.59 to 0.89; P = 0.002) and allocation to levofloxacin (OR: 0.19; 95% CI, 0.04 to 0.87; P = 0.03). Hence, they concluded that although the rate of infection decreased initially, no significant difference existed in both groups at the end of 90 days.

This conclusion was supported by Harms et al. who did a double-blinded randomized control trial on 80 patients [23]. Their inclusion criteria were non-lacunar ischemic stroke, middle cerebral artery (MCA) territory, NIHSS > 11, the start of therapy < 36 hrs after stroke, and n = 80. It was named the PANTHERIS (Preventive Antibacterial Therapy in Acute Ischemic Stroke) trial. As a primary outcome, they concluded that active treatment with moxifloxacin was associated with a lower rate of infection in post-stroke patients. As a secondary outcome, they studied that no difference was present in the clinical outcome after 180 days. Fluoroquinolones are broad-spectrum in nature and cover a lot of bugs but come at a cost of some serious side effects, such as skin reactions and tendonitis. Both studies discussed above, the ESPIAS study and the PANTHERIS trial, concluded that despite the decreasing rate of infections, no long-term benefits were seen with antibiotics prophylaxis [15, 23]. Schwarz et al. used fever as an indicator of infection in 2008 [16]. He used mezlocillin and sulbactam as prophylaxis in ischemic stroke patients who did not indicate infection already. Sixty patients were included (mean: 75 years; median National Institutes of Health Stroke Scale score: 16). Over the first three days, patients in the intervention group showed lower mean body temperatures, as well as lower daily peak temperatures (P = 0.05). Throughout the observation period, 15 of 30 patients in the intervention group and 27 of 30 patients in the conventionally treated group developed an infection (P = 0.05). Stefan et al. believed that in patients with acute severe stroke, prophylactic administration of mezlocillin, plus sulbactam, over four days decreases body temperature, lowers the rate of infection, and may be associated with a better clinical outcome. Few antibiotics are considered to have neuroprotective effects and this was the reason for them being utilized in the prophylactic antibiotic trials. Minocycline and ceftriaxone are considered among them [25-26]. Four trials used oral minocycline therapy as prophylactic antibiotics due to this property [17-19, 24]. According to one of the meta-analyses by Schwarz et al., it was concluded that studies including minocycline therapies aimed at neuroprotection after stroke used a narrower time window than the other studies on the prevention of infection. Within the framework of these studies, the infection was, in fact, not of major concern and was not monitored. Three of the four studies reported an association of minocycline treatment with improved clinical outcomes. However, owing to their small sample size and other methodological problems, these promising results now have to be replicated by a larger phase III trial. Srivastava et al. concluded that minocycline can help reduce the clinical deficits after acute ischemic stroke [17]. Similarly, Amiri-Nikpour et al. also concluded that active treatment with minocycline in ischemic stroke within 24 hours is associated with better outcomes at 90 days [19]. Two large clinical trials were carried out in the recent past [20-21]. The Preventive Antibiotics in Stroke Study (PASS) trial and the STROKE-INF (A Cluster Randomised Trial of Different Strategies of Antibiotic Use to Reduce the Incidence and Consequences of Chest Infection in Acute Stroke Patients with Swallowing Problems, ISRCTN37118456) trial. The PASS trial was reported by Westendrop et al. [20]. In this multi-center, randomized, open-label trial with masked endpoint assessment, patients with acute stroke were randomly assigned to intravenous ceftriaxone at a dose of 2 gm given every 24 hrs intravenously for four days, in addition to stroke unit care or standard stroke unit care without preventive antimicrobial therapy; assignments were made within 24 hrs after symptom onset. The primary endpoint was a functional outcome at three months, defined according to the modified Rankin Scale and analyzed by intention to treat. Secondary outcomes included death, infection rates, antimicrobial use, and length of hospital stay. Between July 6, 2010 and March 23, 2014, a total of 2,550 patients from 30 sites in the Netherlands were included in the study. They concluded that preventive ceftriaxone does not improve functional outcome at three months in adults with acute stroke. The results of their trial did not support the use of preventive antibiotics in adults with acute stroke. Kalra et al. did a prospective, multi-center, cluster-randomized, open-label controlled titled the STROKE_INF trial [21]. A masked endpoint assessment of patients older than 18 years with dysphagia after new stroke recruited from 48 stroke units in the United Kingdom was done. Exclusion criteria were patients with contraindications to antibiotics, preexisting dysphagia, known infections, or who were not expected to survive beyond 14 days. The primary outcome was post-stroke pneumonia in the first 14 days, assessed with both a criteria-based, hierarchical algorithm and by physician diagnosis in the intention-to-treat population. Between April 21, 2008 and May 17, 2014, they randomly assigned 48 stroke units (and 1,224 patients clustered within the units) to the two treatment groups. After the trial was done, they concluded that antibiotic prophylaxis cannot be recommended for the prevention of post-stroke pneumonia in patients with dysphagia after stroke managed in stroke units.

Discussion

The trials that have been discussed above are more of the viewpoint that prophylactic antibiotics may decrease the chance of acute infections but are of no long-term benefit.

Brain injury after ischemic or hemorrhagic stroke leads to the activation of inflammatory mediators in the brain [28]. Some think that these mediators cause clearance of the neuronal debris caused by the injury [29-32]. Whereas others are also of the opinion that inflammation caused so early during the stroke phase can cause further irreplaceable damage to brain cells, and this plays a vital role in the ultimate clinical outcome [33-35]. Brain lesion size has been considered as an independent risk factor of post-stroke immunosuppression and infectious complications [36]. A consequence of immunosuppression has been linked to the increased risk of infection after stroke onset [37]. A few researchers studied that by modifying the post-stroke immunosuppression by giving beta-blockers and hypothalamic-pituitary-adrenal axis blockers, the rate of post-stroke infections and survival was reduced [38-41]. The summary of their clinical trials is given in Table 2. 

**Table 2:** Trials on Immunomodulation in Post-stroke Patients HPA: hypothalamic-pituitary-adrenal axis; INF: interferon; MCAO: middle cerebral artery occlusion; RU486: mifepristone

AUTHOR	STUDY DESIGN	INTERVENTION	CONCLUSION
Amjo Jr. et al. [38]	Clinical trial on rat model with MCAO	Beta-blocker: Propranolol; Alpha1 blocker: Prazosin; Adrenergic receptor blocker: Carvedilol	No effect on stroke outcome; no effect on infarct volume; reduced infarct volume and spleen size
Römer et al. [39]	Clinical trial on mouse model with MCAO	Beta-blocker: Propranolol; HPA blocker: RU486	Both reduced infection rates, reduced infarct volume, and increased long-term survival rates
Liu et al. [40]	Clinical trial on mouse model with MCAO	HPA blocker: RU486; Beta-blocker: Propranolol	Reduction in post-stroke infection rate and improved functional outcome
Mracsko et al. [41]	Clinical trial on mouse model with MCAO	HPA blocker: RU486	Intact INF gamma production by lymphocytes; prevents lymphopenia after stroke

Many animal studies in post-stroke models showed that antibiotics reduced post-stroke infections and mortality [42-43]. Studies that we included in our review had different limitations. One of the major issues with most of the studies was the strict inclusion criteria. For example, patients that were under observation in most studies were suffering from a mild form of stroke. Patients with severe stroke were very few and that could have changed the final outcome. More severe brain injury can lead to worse outcomes and more chances of infections due to a higher degree of immunosuppression. Furthermore, the patients under the study were usually in a high-risk setting and were well-cared-for and well-maintained hygienically. This could have potentially reduced the chances of infections as compared to low-risk settings. The STROKE-INF study had all the dysphagic patients included in the study which is itself a major risk factor for aspiration pneumonia. This could have probably caused bias in the final calculations. Although few researchers tried to do blinded studies to reduce observational bias, most did not. Patients in most studies knew about the intervention made and so did the people intervening. This can severely affect the outcomes. Those who put an effort to perform blinded trials were able to reduce the bias but were not sure how well the secrecy was maintained. The choice of antibiotics can also affect the trial results because the use of broad-spectrum antibiotics may not be cost-effective and can have many side effects as well. The researchers considered this and chose the antibiotics with minimal side effects and maximum safety profiles rather than the most effective one. Chamorro et al. hypothesized that the harmful central nervous system effects of levofloxacin may have been responsible for the negative results in their study [15]. These studies ignored the monitoring of the side effects as well. Therefore, despite all of these limitations and feeling the need to have further experimental trials done, we concluded these results.

## Conclusions

After going through a detailed evaluation of these trials, we concluded that pneumonia and UTIs are the two most common post-stroke infections. The use of prophylactic antibiotics can reduce the incidence of these infections. However, the antibiotics have no long-term benefits, neither in neurological outcomes nor in mortality or morbidity. Minocycline and ceftriaxone are thought to have neuroprotective effects but can be associated with drug resistance if used for a longer time. Good nursing care, good hygienic measures, proper care of dysphagic patients, and minimal use of catheters can reduce the risk of infections as well. Post-stroke infections, especially pneumonia, still present challenges for the clinical management of patients with stroke. Modulation of the immune system by beta-blockers or hypothalamic-pituitary-adrenal (HPA) blockers and reducing post-stroke activation of inflammatory mediators can reduce the neuronal damage and this hypothesis is currently being tested.
